# Comparative Analysis of Image Quality Assessment Between Digital Single-Lens Reflex (DSLR) Camera and Smartphone Camera for Dental Photography

**DOI:** 10.7759/cureus.113907

**Published:** 2026-08-03

**Authors:** Kodali Rajyalakshmi Naga Vennela, Devalla Srihari, Ballullaya Srinidhi V, Bollu Indira Priyadarshini, Devalla Venu Babu, Mette Pooja, Gavini Snigdha, Geddam Pravallika, Mohammed Neeman Ali

**Affiliations:** 1 Conservative Dentistry and Endodontics, St. Joseph Dental College, Eluru, IND

**Keywords:** cielab color space, dental photography, matlab analysis, signal-to-noise ratio, smartphone

## Abstract

Background

The growing sophistication of smartphone cameras has prompted enquiry into their suitability as alternatives to digital single-lens reflex (DSLR) cameras for clinical dental photography. This study compared the image quality of a DSLR camera and two smartphone systems, each fitted with an identical macro lens, for intraoral dental photography using objective colorimetric and signal-to-noise ratio (SNR) metrics.

Methods

Thirty dental students were enrolled in this comparative, cross-sectional study. Standardised intraoral photographs were captured across seven views using three camera systems: a DSLR with a Canon 100 mm macro lens and ring flash (Canon Inc., Tokyo, Japan), a Samsung smartphone (Samsung Electronics Co., Ltd., Suwon, South Korea), and an Apple iPhone (Apple Inc., Cupertino, CA), the latter two fitted with an identical custom Sony 100 mm macro lens and twin flash (Sony Group Corporation, Tokyo, Japan). CIELAB colour space (CIE L*, a*, and b*) values were recorded in Adobe Photoshop (Adobe Inc., San Jose, CA), and the total colour difference (ΔE) was calculated. SNR was computed in MATrix LABoratory (MATLAB) (MathWorks, Natick, MA) from a standardised background region. One-way analysis of variance (ANOVA) and Tukey honestly significant difference (HSD) post hoc tests were applied (significance: p < 0.05).

Results

The DSLR produced the most consistent colour rendition and served as the colour reference standard. The mean ΔE between the DSLR and the smartphones was comparable for the two devices (25.63 ± 8.62 for Samsung; 25.98 ± 9.56 for iPhone; p = 0.486), and both values substantially exceeded the clinically acceptable threshold of ΔE ≤ 2.0. Mean lightness (L*) was markedly lower for the DSLR (25.24) than for the Samsung (47.47) and iPhone (47.59) systems (p < 0.001). A warm, yellow-shifted smartphone bias was most evident in the frontal shade-matching view. SNR was highest for the iPhone, followed by the Samsung, and lowest for the DSLR, a reversal attributable to artificial-intelligence-driven computational noise reduction in the smartphones.

Conclusions

The DSLR remains the reference standard for colour-critical intraoral dental photography. The elevated SNR recorded for the smartphones reflected computational noise reduction rather than superior image fidelity. Neither smartphone achieved clinically acceptable colorimetric agreement with the DSLR, nor did brand materially influence this outcome once the optical configuration was standardised. Standardised calibration protocols warrant further investigation before smartphone systems are adopted for colour-critical applications.

## Introduction

Clinical dental photography has evolved from a supplementary record-keeping tool into a core instrument of evidence-based dental practice. Systematic photographic documentation underpins pre-operative assessment, permits longitudinal monitoring of treatment outcomes, and facilitates communication with dental laboratories engaged in the fabrication of aesthetic restorations. Reproducibility and technical consistency are especially critical when shade determination is involved, as even marginal discrepancies in colour can carry direct consequences for clinical and aesthetic outcomes.

For several decades, the digital single-lens reflex (DSLR) camera, typically configured with a 100 mm macro lens and a ring-flash or twin-flash illumination system, has occupied the position of reference standard in clinical dental photography, owing to its superior sensor sensitivity, precise magnification control, and reliable colour reproduction. Nevertheless, the rapid pace of innovation in mobile imaging technology has compelled a re-examination of this hierarchy. A recent quantitative investigation [1] reported that although a DSLR consistently achieved superior performance across image resolution, magnification fidelity, and colour accuracy, a flagship smartphone demonstrated shade accuracy approaching that of the DSLR, and a separate pilot comparison similarly found that DSLR cameras produced more consistent and reproducible colour values relative to mobile devices [2].

Despite the continued pre-eminence of DSLR systems, the proliferation of multi-lens array architectures, next-generation sensor technologies, and artificial-intelligence-driven computational photography algorithms in contemporary smartphones has begun to erode this advantage [3,4]. When photographs are acquired at a standardised working distance, smartphones have been shown to yield linear dimensional measurements statistically equivalent to those obtained using DSLR cameras [5], and gingival colour and clinical measurements obtained with a smartphone have been reported as comparable to those obtained with a professional camera [6].

Colour accuracy holds particular clinical primacy in Conservative Dentistry, where the precision of shade determination directly affects material selection and the aesthetic success of restorative treatment. The CIELAB colorimetric model, adopted internationally as a standard for colour communication, describes colour using three perceptually uniform axes: L* (lightness), a* (red-to-green), and b* (yellow-to-blue). The total colour difference between two imaging systems is quantified as ΔE, with a threshold of ≤ 2 widely accepted as clinically imperceptible [7,8]. The incorporation of computational image-analysis platforms such as MATrix LABoratory (MATLAB) programming language platform (MathWorks, Natick, MA) has further elevated the objectivity of dental photographic research by providing a reproducible, operator-independent framework for extracting colorimetric parameters and quantifying signal-to-noise ratio (SNR) [9].

The present investigation was designed to provide an objective, multi-parametric assessment of three camera systems, a DSLR with a Canon 100 mm macro lens and ring flash (Canon Inc., Tokyo, Japan), a Samsung smartphone (Samsung Electronics Co., Ltd., Suwon, South Korea), and an iPhone (Apple Inc., Cupertino, CA), both smartphones fitted with an identical custom Sony 100 mm macro lens and twin flash (Sony Group Corporation, Tokyo, Japan), across seven standardised intraoral photographic views, using CIELAB colour space analysis and MATLAB-derived SNR as the primary outcome measures. By imposing a common macro-optic on both smartphones, this design sought to isolate the contribution of the sensor and image-processing pipeline from that of the lens, a confound present in much of the existing literature.

## Materials and methods

Study design and setting

This comparative, cross-sectional clinical study was conducted in the Department of Conservative Dentistry and Endodontics of the teaching dental institution located in Eluru City of Andhra Pradesh, India. The study was approved by the Institutional Ethical Committee (CEC/05/2024) on 28 February 2024, prior to its commencement, and was conducted in accordance with the rules stated in the Declaration of Helsinki. Data collection was carried out over a six-month period, from 1st March 2024 to 31st August 2024. The study was reported in accordance with the STROBE (Strengthening the Reporting of Observational Studies in Epidemiology) guidelines for cross-sectional studies.

Study population and sample size

Thirty dental students (n = 30) were enrolled as study subjects upon obtaining consent and willingness to participate, recruited from the undergraduate dental student pool at the institution. Because each subject was photographed with all three camera systems across seven standardised views, the investigation employed a repeated-measures design that yielded 630 images in total (210 per camera system), with every subject serving as their own control. To detect a large effect (Cohen's f = 0.40) in a three-group one-way ANOVA at α = 0.05 with 80% power, approximately 66 observations (22 per group) were required [10], a threshold comfortably exceeded by the 90 image-sets analysed per parameter.

Camera systems

Images were captured using three imaging systems: (i) a DSLR camera fitted with a Canon 100 mm macro lens and ring flash; (ii) a Samsung A23 model smartphone fitted with a custom-made Sony 100 mm macro lens and twin flash; and (iii) an Apple iPhone 15 model phone fitted with an identical custom-made Sony 100 mm macro lens and twin flash.

Photographic protocol

**Figure 1:**
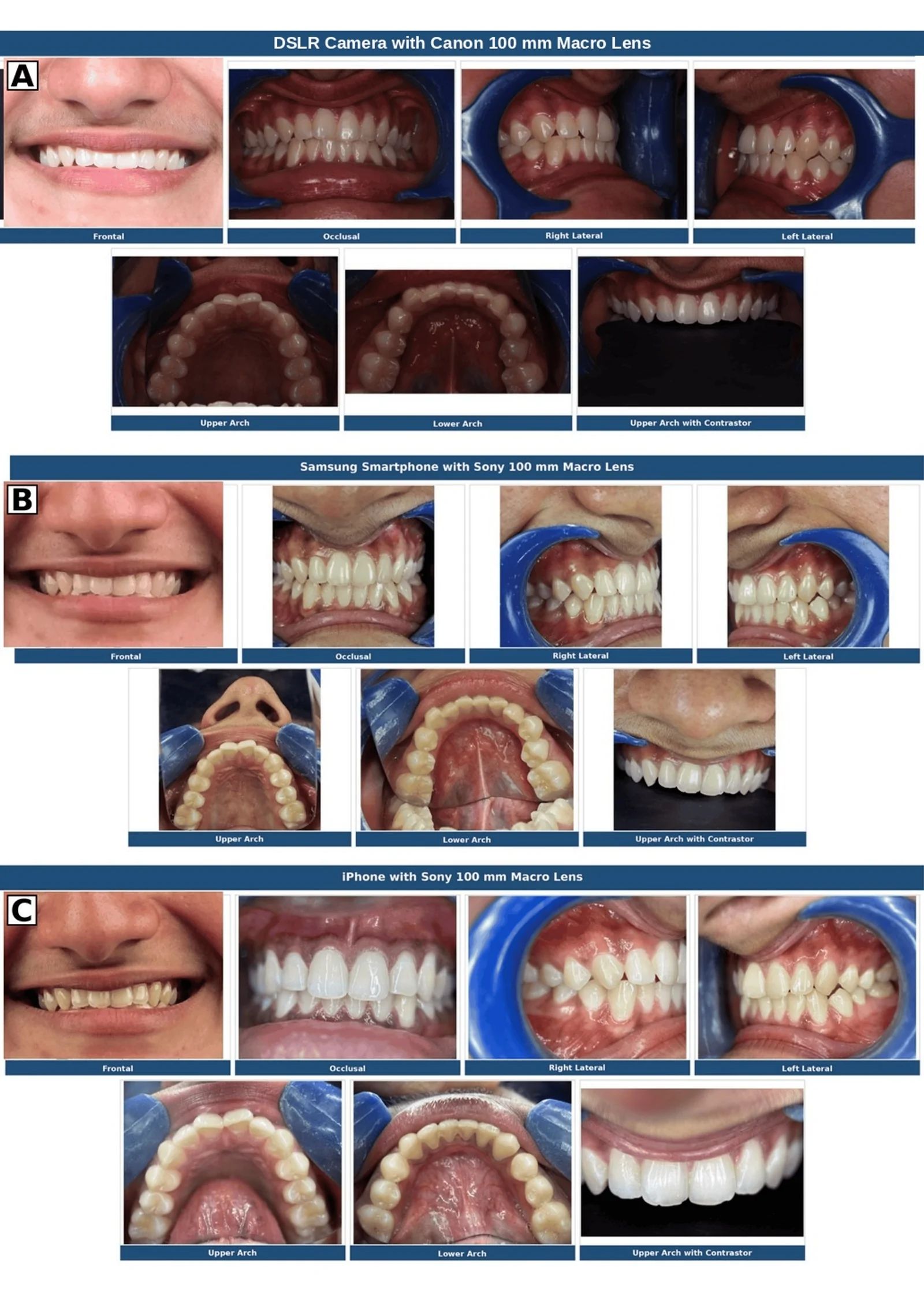
Seven standardised intraoral views captured for a representative subject with each camera system (A) DSLR camera with a Canon 100 mm macro lens and ring flash; (B) Samsung smartphone with a custom Sony 100 mm macro lens and twin flash; (C) iPhone with an identical custom Sony 100 mm macro lens and twin flash. Views (left to right, top to bottom): frontal, occlusal, right lateral, left lateral, upper arch, lower arch, and upper arch with black contrastor. The frontal view has been cropped to the peri-oral region so that the subject's eyes and identifying features are not shown. DSLR: digital single-lens reflex

Seven standardised intraoral views were captured for each subject with each camera system: frontal, occlusal, right lateral, left lateral, upper arch, lower arch, and upper arch with a black contrastor (Figure 1). Cheek retractors, contrastors, and intraoral mirrors were used as required for each view. The DSLR was operated using fixed manual exposure settings; automatic exposure could not be fully disabled on the smartphone devices, and this is acknowledged as a limitation.

Colorimetric (CIELAB) analysis

**Figure 2:**
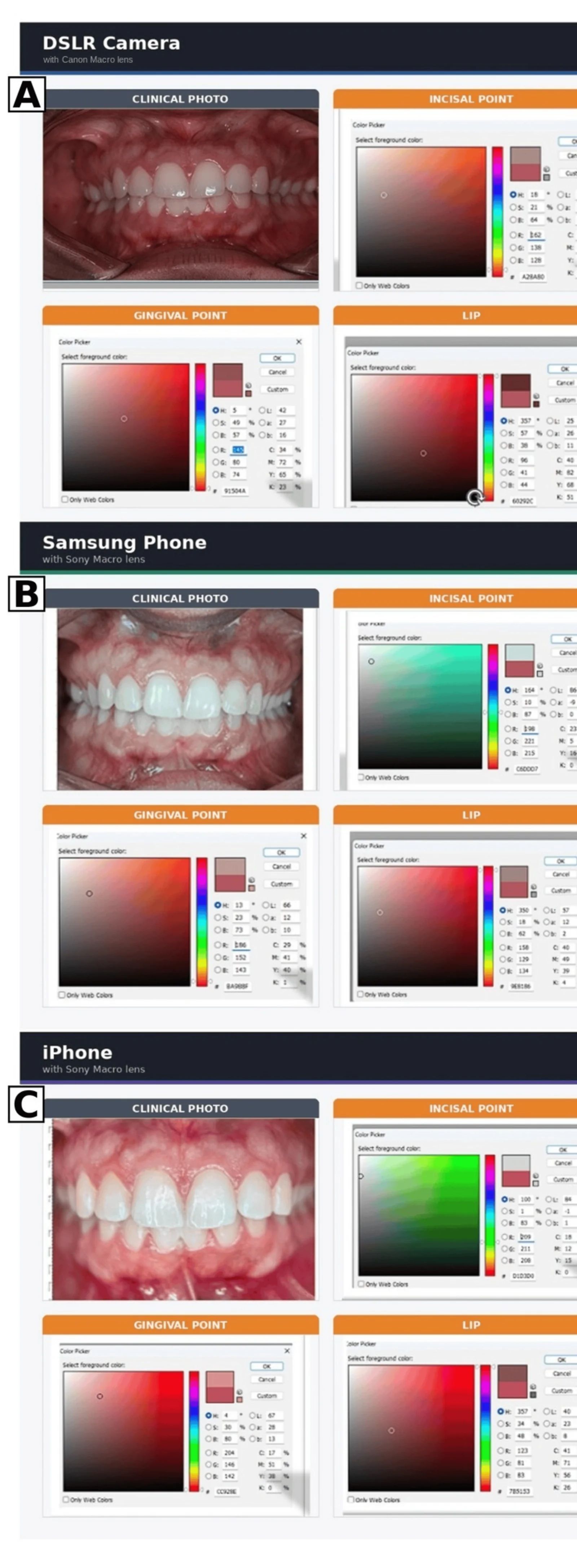
CIELAB colour sampling and colour picker analysis of intraoral photographs obtained using a digital single-lens reflex (DSLR) camera and smartphone cameras (A) Intraoral photograph captured using a DSLR camera fitted with a Canon macro lens and ring flash, showing CIELAB colour sampling at the incisal, gingival, and lip regions using the Adobe Photoshop colour picker. (B) Intraoral photograph captured using a Samsung smartphone fitted with a custom-made Sony macro lens and twin flash, demonstrating the corresponding CIELAB colour sampling at the incisal, gingival, and lip regions. (C) Intraoral photograph captured using an Apple iPhone fitted with a custom-made Sony macro lens and twin flash, demonstrating the corresponding CIELAB colour sampling at the incisal, gingival, and lip regions. The displayed colour picker values were used to obtain the CIELAB coordinates (L*, a*, and b*) for subsequent colour analysis and comparison among the imaging systems.

Each image was opened in Adobe Photoshop (Adobe Inc., San Jose, CA) and converted to Lab Color mode. CIE L*, a*, and b* values were sampled at three standardised points per view: an incisal point, a gingival point, and a lip point, using the colour sampler tool, and the mean of the three readings was recorded as the representative colour value for that view (Figure 2). Identical sampling points were used across all three camera systems (Figures 2A-2C). The total colour difference was calculated as $\Delta E = \sqrt{(\Delta L^{*})^{2} + (\Delta a^{*})^{2} + (\Delta b^{*})^{2}}$.

Signal-to-noise ratio analysis

SNR was computed in MATLAB. For each image, the red, green, and blue channels were separated and converted to double precision, and a standardised 30 × 30 pixel uniform background region on the cheek-retractor surface was selected. The mean pixel intensity (μ) and standard deviation (σ) were calculated for each channel, and SNR was derived in decibels as SNR = 20 × log₁₀(μ/σ). Per-channel values were averaged to yield a single SNR value per image.

Statistical analysis

All statistical analyses were performed using IBM SPSS Statistics for Windows, Version 29.0 (IBM Corp., Armonk, NY). Normality was assessed using the Shapiro-Wilk test. One-way ANOVA was used to compare CIE L*, a*, b*, and SNR values across the three camera systems, with Tukey honestly significant difference (HSD) post hoc tests applied where ANOVA identified significant differences. Paired comparisons between the two smartphone systems used the paired t-test and Wilcoxon signed-rank test. Statistical significance was defined at p < 0.05.

## Results

All 630 images were successfully captured and analysed, with no exclusions. The findings are presented below.

Colorimetric (CIELAB) findings

The DSLR recorded consistently lower mean L* values than both smartphone systems across all views except the frontal view, where no difference was observed (p = 0.569). Averaged across all seven views, mean L* was 25.24 for the DSLR compared with 47.47 for the Samsung and 47.59 for the iPhone, a difference confirmed as statistically significant on one-way ANOVA in six of seven views (p < 0.001 for all; Table 1). Post hoc pairwise comparisons are presented in Table 2.

**Table 1:** Mean CIE L* (lightness) values (mean ± SD) by camera system and photographic view Values are mean ± standard deviation. n = 30 subjects per camera system per view (210 images per camera in total). Between-group differences were tested using one-way analysis of variance (ANOVA). L* = lightness (range 0-100; 0 = perfect black, 100 = perfect white). DSLR: digital single-lens reflex

View	n/camera	DSLR L*	Samsung L*	iPhone L*	F-value	p-value
Frontal	30	31.06 ± 4.39	29.04 ± 7.72	30.30 ± 9.25	0.568	0.569
Occlusal	30	27.35 ± 5.55	51.21 ± 1.99	50.88 ± 2.00	434.283	< 0.001
Right lateral	30	30.89 ± 7.39	51.85 ± 5.90	49.71 ± 6.50	90.948	< 0.001
Left lateral	30	28.98 ± 5.71	50.99 ± 5.70	50.86 ± 4.30	173.106	< 0.001
Upper arch	30	20.03 ± 5.54	49.64 ± 3.22	51.27 ± 4.61	446.971	< 0.001
Lower arch	30	21.12 ± 4.80	49.70 ± 3.21	51.49 ± 2.47	677.792	< 0.001
Contrastor	30	17.21 ± 5.77	49.78 ± 4.13	48.59 ± 5.90	360.511	< 0.001

**Table 2:** Post hoc pairwise comparisons of mean CIE L* values (Tukey HSD) by photographic view Mean difference = second group minus first group (computed from the group means in Table 1); 95% CI and adjusted p values are from Tukey HSD post hoc testing. CI: confidence interval; DSLR: digital single-lens reflex

View	Comparison	Mean difference	95% CI	p value
Frontal	DSLR vs Samsung	-2.02	-6.58 to 2.54	0.545
	DSLR vs iPhone	-0.76	-5.31 to 3.80	0.918
	Samsung vs iPhone	1.26	-3.30 to 5.82	0.787
Occlusal	DSLR vs Samsung	23.85	21.64 to 26.07	< 0.001
	DSLR vs iPhone	23.52	21.31 to 25.74	< 0.001
	Samsung vs iPhone	-0.33	-2.55 to 1.88	0.932
Right lateral	DSLR vs Samsung	20.95	16.88 to 25.03	< 0.001
	DSLR vs iPhone	18.82	14.74 to 22.90	< 0.001
	Samsung vs iPhone	-2.13	-6.21 to 1.94	0.428
Left lateral	DSLR vs Samsung	22.01	18.77 to 25.26	< 0.001
	DSLR vs iPhone	21.89	18.64 to 25.13	< 0.001
	Samsung vs iPhone	-0.13	-3.38 to 3.12	0.995
Upper arch	DSLR vs Samsung	29.62	26.81 to 32.42	< 0.001
	DSLR vs iPhone	31.24	28.44 to 34.05	< 0.001
	Samsung vs iPhone	1.62	-1.18 to 4.43	0.355
Lower arch	DSLR vs Samsung	28.63	26.42 to 30.84	< 0.001
	DSLR vs iPhone	30.38	28.17 to 32.59	< 0.001
	Samsung vs iPhone	1.75	-0.46 to 3.96	0.148
Contrastor	DSLR vs Samsung	32.57	29.29 to 35.85	< 0.001
	DSLR vs iPhone	31.37	28.09 to 34.65	< 0.001
	Samsung vs iPhone	-1.19	-4.47 to 2.09	0.662

The overall mean colour difference (ΔE) between the DSLR and the smartphones was comparable for the two devices, 25.63 ± 8.62 for DSLR vs Samsung and 25.98 ± 9.56 for DSLR vs iPhone (Table 3), with no significant difference between them on paired comparison (p = 0.486). Both values lay well above the clinically imperceptible threshold of ΔE ≤ 2.0. The a* (red-green) axis was 13.21, 10.74, and 13.43 for the DSLR, Samsung, and iPhone respectively, and the b* (yellow-blue) axis was 6.58, 4.21, and 6.40, respectively, both differing significantly on one-way ANOVA (a*: F = 18.517, p < 0.001; b*: F = 16.782, p < 0.001). In the frontal shade-matching view specifically, both smartphones exhibited a warm, yellow-shifted bias relative to the DSLR (b* +2.94 for Samsung and +7.57 for iPhone, vs -1.31 for the DSLR).

**Table 3:** Overall mean colour difference (ΔE) between the DSLR and each smartphone system, averaged across all seven photographic views DSLR: digital single-lens reflex; SD: standard deviation; ΔE: total colour difference

Comparison	Mean ΔE ± SD	Interpretation
DSLR vs Samsung	25.63 ± 8.62	Exceeds clinical threshold (ΔE ≤ 2.0)
DSLR vs iPhone	25.98 ± 9.56	Exceeds clinical threshold (ΔE ≤ 2.0)

Signal-to-noise ratio findings

Contrary to the colorimetric findings, the iPhone recorded the highest mean SNR, followed by the Samsung, with the DSLR recording the lowest SNR across the seven views. One-way ANOVA confirmed statistically significant inter-system differences in six of the seven views (p < 0.001), the frontal view being the exception (F = 1.177, p = 0.313; Table 4). The most pronounced difference occurred in the upper arch with contrastor view (F = 83.092, p < 0.001). Tukey HSD post hoc analysis localised these differences to all three pairwise comparisons in the upper-arch view, while in the contrastor view the DSLR differed significantly from both smartphones, which did not differ from each other (Table 5).

**Table 4:** Mean signal-to-noise ratio (SNR, dB) by camera system and photographic view, with one-way ANOVA SNR values are mean ± SD in decibels (n = 30 per camera per view). The frontal view showed no significant inter-system difference (F = 1.177, p = 0.313); the upper arch with contrastor showed the largest (F = 83.092, p < 0.001). Between-group differences were tested using one-way ANOVA (Analysis of Variance). DSLR: digital single-lens reflex; SD: standard deviation; SNR: signal-to-noise ratio

View	DSLR SNR	Samsung SNR	iPhone SNR	F value	p value
Frontal	4.03 ± 1.24	4.16 ± 1.80	4.74 ± 2.50	1.177	0.313
Occlusal	6.62 ± 1.18	7.53 ± 1.61	8.55 ± 0.60	19.344	< 0.001
Right lateral	6.42 ± 1.28	8.18 ± 1.22	8.54 ± 0.62	32.883	< 0.001
Left lateral	6.85 ± 1.29	7.94 ± 1.32	8.70 ± 0.70	19.787	< 0.001
Upper arch	6.12 ± 1.16	7.37 ± 1.09	8.73 ± 0.90	45.742	< 0.001
Lower arch	6.61 ± 1.19	7.43 ± 1.31	8.29 ± 1.04	14.986	< 0.001
Contrastor	3.50 ± 0.74	6.36 ± 1.57	7.01 ± 0.88	83.092	< 0.001

**Table 5:** Post hoc pairwise comparisons of mean SNR values (Tukey HSD) by photographic view Mean difference = second group minus first group (from the SNR means in Table 4); p-values are adjusted (Tukey HSD). All three pairwise comparisons differed significantly in the upper-arch view; in the contrastor view, the DSLR differed significantly from both smartphones, which did not differ from each other (p = 0.067). DSLR: digital single-lens reflex; SNR: signal-to-noise ratio

View	Comparison	Mean difference	p-value
Frontal	DSLR vs Samsung	0.13	0.959
	DSLR vs iPhone	0.71	0.322
	Samsung vs iPhone	0.58	0.475
Occlusal	DSLR vs Samsung	0.91	0.012
	DSLR vs iPhone	1.93	< 0.001
	Samsung vs iPhone	1.02	0.004
Right lateral	DSLR vs Samsung	1.76	< 0.001
	DSLR vs iPhone	2.12	< 0.001
	Samsung vs iPhone	0.36	0.413
Left lateral	DSLR vs Samsung	1.09	0.001
	DSLR vs iPhone	1.85	< 0.001
	Samsung vs iPhone	0.76	0.032
Upper arch	DSLR vs Samsung	1.25	< 0.001
	DSLR vs iPhone	2.61	< 0.001
	Samsung vs iPhone	1.36	< 0.001
Lower arch	DSLR vs Samsung	0.82	0.025
	DSLR vs iPhone	1.68	< 0.001
	Samsung vs iPhone	0.86	0.017
Contrastor	DSLR vs Samsung	2.86	< 0.001
	DSLR vs iPhone	3.51	< 0.001
	Samsung vs iPhone	0.65	0.067

Table 6 describes the paired comparison of the two smartphone systems on the colorimetric parameters, using the paired t-test on the raw per-subject data (n = 210 paired observations), where it was observed that L* did not differ significantly between the two phones (p = 0.822), whereas a* and b* did (both p < 0.001). The overall ΔE relative to the DSLR was comparable for the two devices (t = -0.699, p = 0.486), confirming that smartphone brand did not materially affect agreement with the reference standard. ΔE values were computed from the CIE L*, a*, and b* coordinates.

**Table 6:** Paired comparison of the two smartphone systems (Samsung vs iPhone): paired t-test Comparisons were made using the paired t-test on the raw per-subject data (n = 210 paired observations per parameter); the assumptions for parametric testing were met on Shapiro-Wilk testing. Mean L* did not differ significantly between the two smartphones (p = 0.822), whereas mean a* and b* differed significantly (both p < 0.001). The overall ΔE relative to the DSLR was comparable for the two devices (p = 0.486). DSLR: digital single-lens reflex; ΔE: total colour difference

Parameter	Samsung (mean)	iPhone (mean)	t value	p (t-test)
Mean L*	47.47	47.59	-0.225	0.822
Mean a*	10.74	13.43	-7.145	< 0.001
Mean b*	4.21	6.4	-4.843	< 0.001
Mean ΔE vs DSLR	25.63	25.98	-0.699	0.486

## Discussion

Clinical dental photography is employed across virtually every dental discipline for diagnosis, treatment planning, laboratory communication, and patient education, and the reliability of these functions is contingent upon the fidelity with which the camera reproduces the true colour and form of the dentition [11,12]. The present study was designed to provide a quantitative, operator-independent comparison of three contemporary camera systems using two complementary objective metrics, the CIELAB colour space and MATLAB-derived SNR, consistent with the analytical framework adopted in prior multi-camera comparisons [2].

The selection of the DSLR as the colour reference standard is well supported by the literature. Calibrated DSLR cameras have previously achieved the lowest mean ΔE of any tested imaging system following polynomial regression calibration [7], and DSLR systems have repeatedly been affirmed as superior for colour-critical intraoral photography [13], producing the most neutral colour rendition among compared systems [2]. The principal advantage of the DSLR resides in its capacity to record incident light in an approximately linear fashion, without the aggressive tone-mapping and adaptive white-balance corrections that characterise consumer imaging devices.

The overall mean ΔE recorded here for both smartphone systems (25.63 and 25.98) substantially exceeded not only the clinical perceptibility threshold of ΔE ≤ 2.0 but also the acceptability thresholds reported for colour matching using digital photographic techniques in restorative dentistry [14], indicating that neither smartphone reproduced dentition colour in a manner acceptable for shade-critical applications when referenced against the DSLR. This finding aligns with reports that mobile devices systematically overestimate tooth luminosity [2] and, in shade-matching views, exhibit a warm colour bias attributable to adaptive white-balance and aesthetic-enhancement algorithms [15,16].

A distinctive methodological feature of the present investigation was the use of an identical macro lens on both smartphone systems, intended to neutralise the geometric and magnification differences that confound many smartphone-versus-DSLR comparisons. A DSLR fitted with a 100 mm macro lens is known to produce proportionally accurate images consistent with true anatomical dimensions, whereas native smartphone optics introduce perspective distortion at conventional working distances [5], and measurable barrel distortion has similarly been documented in smartphones used at close range [17]. The finding that the two smartphones did not differ significantly in colorimetric agreement with the DSLR (p = 0.486), yet both differed greatly from the DSLR, suggests that the dominant determinant of colour error in contemporary smartphone dental photography is the computational image-processing pipeline rather than the optical front end, a refinement of the existing literature, which has often attributed smartphone variability to lens differences alone. This contrasts with brand-specific performance differentials reported elsewhere [18], plausibly because standardising the lens across devices in the present study suppressed the inter-brand optical variation that typically drives such differences.

The a* axis discrepancy observed between the two smartphones, with the Samsung system underrepresenting the red chromatic component relative to both the DSLR and the iPhone, carries particular relevance for the assessment of gingival health, since gingival erythema manifests as an elevation in the positive a* component of tissue colour [19]. A camera system that systematically underestimates a* risks underrepresenting the degree of gingival inflammation in photographic records, illustrating that aggregate ΔE statistics can mask clinically relevant axis-specific biases.

Contrary to the colorimetric findings, SNR was highest for the iPhone and lowest for the DSLR, a reversal that should not be interpreted as superior smartphone image quality. Contemporary flagship smartphones construct each image through a computational pipeline incorporating multi-frame stacking, high-dynamic-range tone mapping, and machine-learning-based denoising [15,20-23]. Multi-frame stacking suppresses random photonic noise, which mathematically inflates SNR, while simultaneously smoothing fine surface detail and compressing tonal variation, the same processes responsible for the large ΔE observed in this study. The DSLR's comparatively larger sensor and unprocessed linear output preserve true colour fidelity at the cost of a lower calculated SNR, consistent with prior reports that DSLR systems record lower image noise than mobile-phone equivalents despite this apparent SNR disadvantage [2,24-25].

Limitations

Several limitations warrant acknowledgement. Automatic exposure could not be fully disabled on the smartphone devices, meaning the absolute L* values reported are not directly comparable to spectrophotometric reference data, although the internal validity of the relative inter-device comparison is preserved because every image was sampled using an identical region-of-interest protocol. Colour sampling was performed manually in Photoshop at three points per view rather than across the full image, and the study cohort was drawn from a homogeneous student population, which may limit generalisability to more diverse patient presentations. In addition, although each subject served as their own control across the three camera systems, between-camera comparisons were analysed using one-way ANOVA; a repeated-measures model could be applied in future work to exploit this paired structure. Future investigations incorporating standardised calibration protocols, such as grey-card white-balance correction and cross-polarisation, are warranted to determine whether the colorimetric performance gap between DSLR and smartphone systems can be reduced to clinically acceptable levels.

## Conclusions

Within the limitations of the present study, the DSLR camera demonstrated superior and more consistent colorimetric accuracy than both smartphone systems for intraoral dental photography and accordingly served as the colour reference standard. Neither smartphone achieved clinically acceptable colour agreement with the DSLR, and the two devices performed comparably to one another, indicating that smartphone brand was not a decisive factor once the optical configuration was standardised through a common macro lens. The higher SNR recorded for the smartphones reflected computational noise reduction rather than superior image quality. The DSLR therefore remains the preferred instrument for colour-critical intraoral dental photography, particularly for shade selection, while smartphone cameras fitted with a standardised macro lens may serve a supportive role in general clinical documentation and patient education pending validation of standardised calibration protocols.
