# Correction: Comparative Analysis of Image Quality Assessment Between Digital Single-Lens Reflex (DSLR) Camera and Smartphone Camera for Dental Photography

**DOI:** 10.7759/cureus.c478

**Published:** 2026-09-09

**Authors:** Kodali Rajyalakshmi Naga Vennela, Devalla Srihari, Ballullaya Srinidhi V, Bollu Indira Priyadarshini, Devalla Venu Babu, Mette Pooja, Gavini Snigdha, Geddam Pravallika, Mohammed Neeman Ali

**Affiliations:** 1 Conservative Dentistry and Endodontics, St. Joseph Dental College, Eluru, IND

The title of this article has been revised to the following: Comparative Analysis of Image Quality Assessment Between Digital Single-Lens Reflex (DSLR) Camera and Smartphone Camera for Dental Photography. The original title incorrectly identified the camera as a Digital Signal-to-Noise Ratio (SNR) Camera.

